# Crucial Role of Lymphocytes in the Therapeutic Efficacy of Dendritic Cell Immunotherapy for Advanced Hepatocellular Carcinoma

**DOI:** 10.3390/ijms27188354

**Published:** 2026-09-19

**Authors:** Wei-Chen Lee, Yin Lai, Hao-Chien Hung, Jin-Chiao Lee, Yu-Chao Wang, Chih-Hsien Cheng, Tsung-Han Wu, Chen-Fang Lee, Ting-Jung Wu, Hong-Shiue Chou, Kun-Ming Chan

**Affiliations:** 1 Division of Liver and Transplantation Surgery, Department of General Surgery, Chang-Gung Memorial Hospital, 5, Fu-Hsing Street, Linkou, Taoyuan 333423, Taiwan; mr1709@cgmh.org.tw (Y.L.); mp0616@cgmh.org.tw (H.-C.H.); jinchiao@cgmh.org.tw (J.-C.L.); awuang726@gmail.com (Y.-C.W.); chengcchj@cgmh.org.tw (C.-H.C.); wutsunghan@gmail.com (T.-H.W.); lee5310@cgmh.org.tw (C.-F.L.); wutj5056@gmail.com (T.-J.W.); chouhs@cgmh.org.tw (H.-S.C.); chankunming@cgmh.org.tw (K.-M.C.); 2 College of Medicine, Cheng-Gung University, Taoyuan 333423, Taiwan

**Keywords:** dendritic cell, hepatocellular carcinoma, T-lymphocytes, neutrophil-to-lymphocyte ratio, immunotherapy

## Abstract

At present, immune checkpoint inhibitors (ICIs) and tyrosine kinase inhibitors (TKIs) are used to treat advanced hepatocellular carcinoma (HCC), with limited therapeutic effects. Dendritic cell (DC) immunotherapy may be a feasible option following ICI/TKI therapy to increase patient survival. Seventy patients with HCC who received DC therapy were included in this study. DCs were propagated from peripheral blood monocytes and pulsed with tumor lysate. The phenotypes of peripheral white blood cells were analyzed prior to and after DC therapy. Among the 70 patients examined, 11 (15.7%) had an objective response (group A), 26 (37.1%) had stable disease and minor regression (group B), 21 (30.0%) had stable disease and minor progression (group C), and 12 (17.1%) had progressive disease (group D). Prior to DC therapy, patients in group A had a higher frequency of CD8^+^ T-cells and a lower neutrophil-to-lymphocyte ratio than patients in the other groups. After DC therapy, patients in groups A and B exhibited a significant increase or tendency to increase in the frequencies of CD3^+^, CD4^+^, and CD8^+^ T-cells. The 1-, 2-, and 3-year survival rates were 90.9%, 81.8%, and 63.3%, respectively, for group A patients; 76.9%, 50.5%, and 26.9% for group B patients, which were significantly better than the rates of 42.9%, 9.5%, and 4.8% for group C patients and 25%, 8.3%, and 0% for group D patients (*p* < 0.001). In conclusion, DC therapy following ICI/TKI therapy is feasible for patients with advanced HCC. Patients with objective or minor responses to DC therapy exhibited an increased frequency of T-cells and improved survival.

## 1. Introduction

Hepatocellular carcinoma (HCC) is the most common primary malignancy of the liver and one of the leading causes of cancer-related deaths. Various therapeutic modalities are used to treat HCC, such as liver transplantation, surgical resection, local ablation, transcatheter arterial chemoembolization, radiotherapy, molecular targeting therapy, and immunotherapy [1,2,3]. According to the Barcelona Clinic Liver Cancer (BCLC) staging and treatment strategy, the choice of treatment depends on the tumor stage and liver functional preservation [4]. For advanced-stage HCC, a combination of immune checkpoint inhibitors (ICIs) and tyrosine kinase inhibitors (TKIs) or dual ICIs has become the first-line treatment [5,6,7]. Under the combination of ICIs and TKIs or dual ICIs, the objective response rate can approach 30%. These treatments have demonstrated promising outcomes in patients with advanced HCC, with patients who achieve an objective response potentially experiencing longer overall survival. However, a substantial proportion of patients fail to respond to ICI/TKI or dual ICI treatments, and their prognosis remains poor. Moreover, immunotherapy-related adverse effects (irAEs) remain a major concern as high-grade irAEs can be life-threatening [8,9,10]. In an effort to improve treatment outcomes for patients with advanced HCC who are unresponsive to ICI/TKI treatment and unable to tolerate its adverse effects, dendritic cell (DC) immunotherapy has been introduced as an alternative therapeutic approach.

Ex vivo dendritic cell (DC) immunotherapy is a safe treatment option for patients who do not respond to ICI/TKI treatment. Dendritic cells (DCs) are the most potent antigen-presenting cells that express high levels of co-stimulatory molecules and major histocompatibility complex (MHC) classes I and II. When they encounter antigens, they capture antigens and process them, undergo maturation, and migrate to T-cell-dependent areas of secondary lymphoid organs, where they activate T-cells. Cancer cells develop as a consequence of enhanced or aberrant expression of oncogenes or loss of tumor suppressor genes. Genetic changes in cancer cells can induce the expression of new antigens. Theoretically, dendritic cells can recognize foreign antigens and trigger T-cells to eradicate tumors. Clinically, ex vivo DCs pulsed with tumor-specific or tumor-associated antigens can be used for tumor-specific immunotherapies [11,12,13]. In this study, peripheral blood monocyte (PBMC)-derived DCs pulsed with autologous tumor lysate were used to treat advanced HCC.

Dendritic cells have been used to treat various cancers, with promising effects reported. For patients with advanced HCC, the clinical benefits of DC immunotherapy have been observed in several clinical studies [14,15,16]. However, the therapeutic responses and clinical benefits of DC immunotherapy remain indeterminate. In this study, we investigated alterations in immune cells and their association with clinical responses to DC immunotherapy in patients with advanced HCC.

## 2. Results

### 2.1. Characteristics of Patients and Responses to DC Therapy

Among the 70 patients examined in this study, 54 (77.1%) were male, and 16 were female. A total of 10 (14.3%) patients had late-stage B disease, while 60 (85.7%) patients had stage C disease. All patients received three courses of autologous DC therapy. Prior to DC therapy, 54 (77.1%) patients received TKI treatment, and 44 (62.9%) patients received ICI treatment. According to the mRECIST criteria, 11 (15.7%) patients were classified as having an objective response (group A), 26 (37.1%) as having stable disease with minor regression (group B), 21 (30%) as having stable disease with minor progression (group C), and 12 (17.1%) as having progressive disease (group D). The number of DCs yielded for therapy did not differ among the four groups. The characteristics of the four groups of patients are listed in Table 1. Of the 10 BCLC late-stage B patients, 5 patients were assigned to group A, 3 to group B, 1 to group C, and 1 to group D (*p* = 0.013). Of the 44 patients who received ICI treatment, 20 exhibited ICI resistance, with the remaining 24 completing the planned ICI treatment before starting DC therapy. Among the 24 patients with planned ICI treatment, 6 patients were assigned to group A, 12 to group B, 3 to group C, and 3 to group D (*p* = 0.007). After DC therapy, three patients in group A and one patient in group B underwent conversion to surgical treatment (Table 1).

### 2.2. Overall Survival

**Figure 1:**
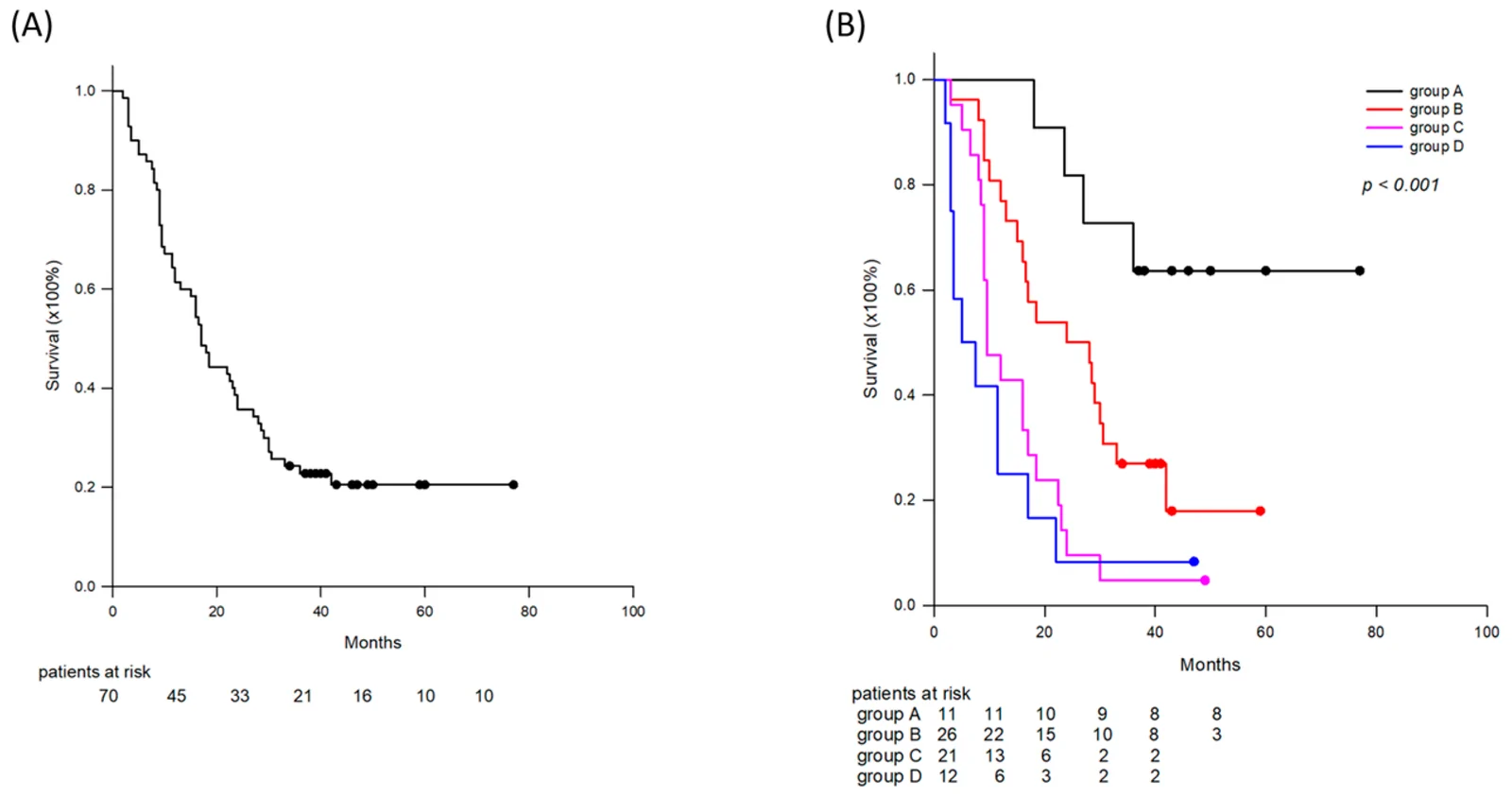
Kaplan–Meier survival curves for the patients. (**A**) The 1-, 2-, and 3-year survival rates were 61.4%, 35.7%, and 22.8%, respectively, for all 70 patients. (**B**) The 1-, 2-, and 3-year survival was 90.9%, 81.8%, and 63.3%, respectively, for group A patients; 76.9%, 50.5%, and 26.9% for group B patients; 42.9%, 9.5%, and 4.8% for group C patients; and 25%, 8.3%, and 0 for group D patients (*p* < 0.001).

Among all 70 patients, the median survival was 17.0 (95% confidence interval (CI): 13.1–19.9) months. The 1-, 2-, and 3-year survival rates were 61.4%, 35.7%, and 22.8%, respectively (Figure 1A). Survival was further analyzed according to responses after DC therapy. The median overall survival of patients in group A was not reached. The median survival was 24.0 (95% CI: 9.6–38.4) months for group B patients, 9.5 (95% CI: 6.1–12.9) months for group C, and 5.0 (% CI: −1.8–11.8) months for group D. The 1-, 2-, and 3-year survival rates were 90.9%, 81.8%, and 63.3%, respectively, for group A patients; 76.9%, 50.0%, and 26.9% for group B; 42.9%, 9.5%, and 4.8% for group C; and 25%, 8.3%, and 8.3% for group D, respectively (*p* < 0.001, Figure 1B). Overall survival was significantly better in group A than in groups B (*p* = 0.033), C (*p* < 0.001), and D (*p* < 0.001). The overall survival of group B patients was better than that of patients in groups C (*p* = 0.016) and D (*p* = 0.021). The overall survival between groups C and D did not differ significantly (*p* = 0.29).

### 2.3. Immune Cell Analysis Prior to DC Therapy

**Figure 2:**
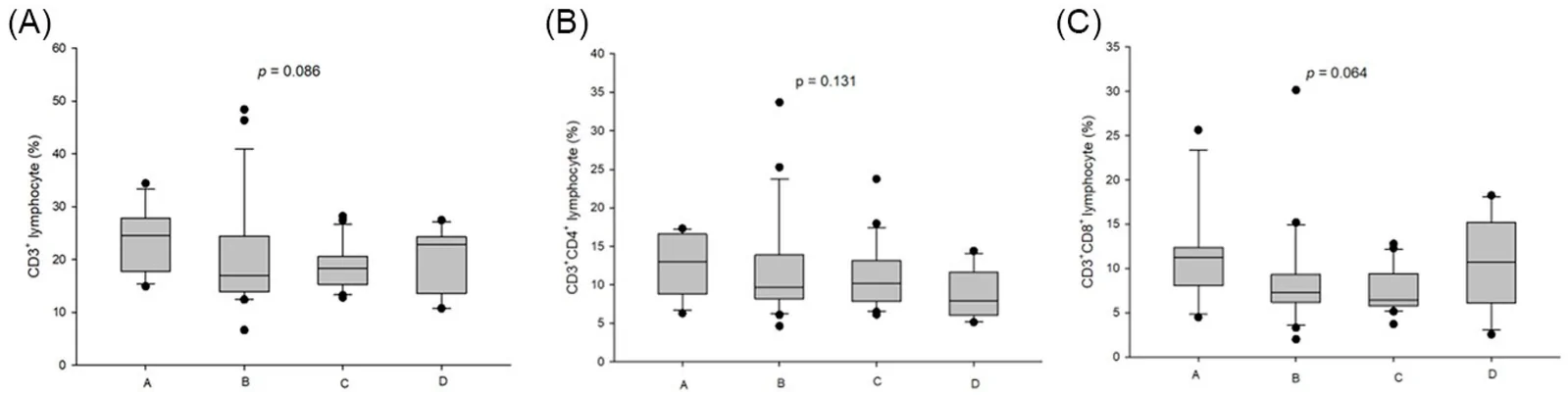
The frequencies of T-cells in the four groups (abscissa A to D) before DC immunotherapy. (**A**) Group A patients tended to have a higher frequency of CD3^+^ T-cells than those in the other groups (median (interquartile range), 24.5 (17.8–27.8)% versus 17.0 (13.9–24.5)%, 18.3 (15.3–20.6)%, and 22.9 (13.6–24.4)%, *p* = 0.086). (**B**) The frequency of CD4^+^ T-cells did not differ among the four groups. (**C**) The frequency of CD8^+^ T-cells in group A patients tended to be higher than in other groups (11.3 (8.1–12.4)% versus 7.3 (6.2–9.3)%, 6.4 (5.8–9.4)%, and 10.7 (6.1–15.2)%, *p* = 0.064).

To determine whether the immune cell frequency in peripheral blood can predict responses after DC therapy, 1 mL of peripheral blood was withdrawn and stained with a panel of cell surface antibodies to analyze the frequencies of T-cells, regulatory T-cells, antigen-presenting cells, and myeloid-derived suppressor cells. The results revealed that group A patients tended to have a higher frequency of CD3^+^ T-cells than those in the other groups (median (interquartile), 24.5 (17.8–27.8)% versus 17.0 (13.9–24.5)%, 18.3 (15.3–20.6)%, and 22.9 (13.6–24.4)%, *p* = 0.086). The frequency of CD8^+^ T-cells in group A patients was also higher than that in the other groups (11.3 (8.1–12.4)% versus 7.3 (6.2–9.3)%, 6.4 (5.8–9.4)%, and 10.7 (6.1–15.2)%, *p* = 0.064) (Figure 2). The frequencies of CD3^+^Foxp3^+^ regulatory T-cells, HLADR^+^CD11c^−^CD123^+^ plasmacytoid cells, and HLADR^−^CD33^+^ myeloid-derived suppressor cells did not differ among the four groups (Table 2).

### 2.4. Neutrophil-to-Lymphocyte Ratio (NLR) Prior to DC Therapy

**Figure 3:**
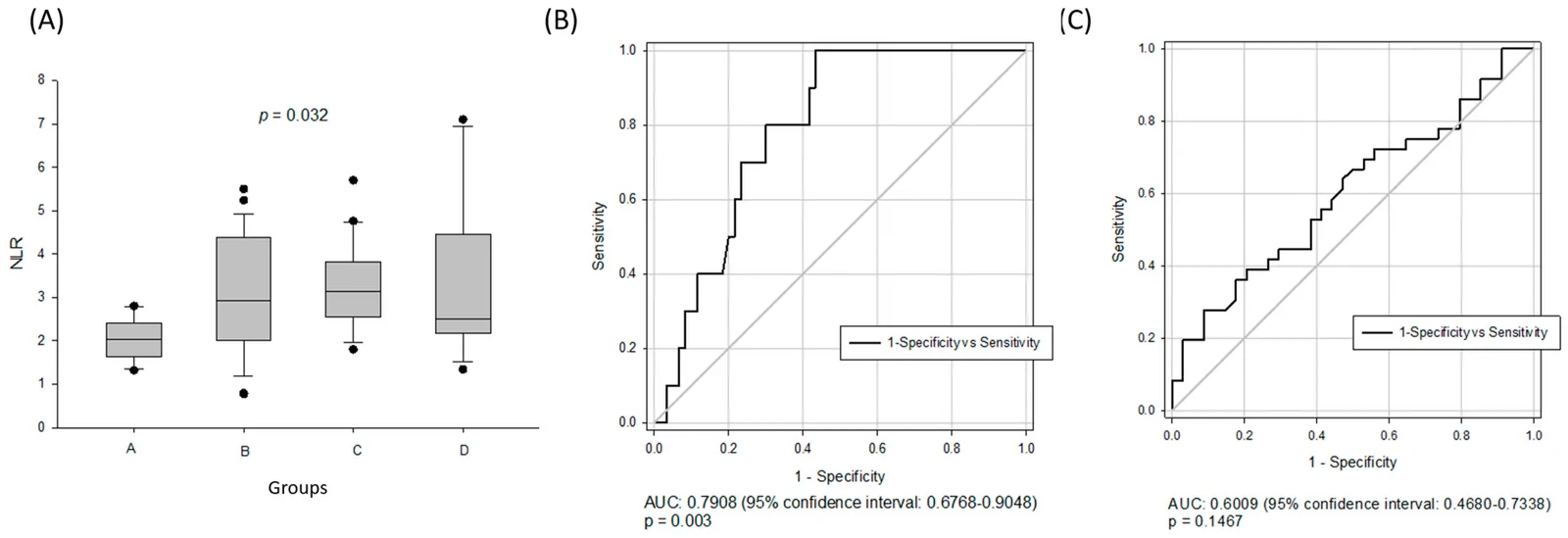
NLR in the four groups (abscissae A to D) of patients before DC immunotherapy. (**A**) Group A patients had a lower NLR than group B, C, and D patients before DC immunotherapy (2.04 ± 0.48 versus 3.12 ± 1.34, 3.23 ± 1.04, and 3.43 ± 1.84, *p* = 0.032). (**B**) Following ROC curve analysis, the area under the ROC for group A with objective response was 0.7908 (*p* = 0.003), and the best cut-off point prior to DC therapy was 2.835 (sensitivity: 1.000 and 1-specificity: 0.5667). (**C**) When the NLR was analyzed for group A+B (objective response and minor response) patients, the area under the ROC curve was 0.6009 (*p* = 0.147).

Differential counts of peripheral white blood cells were measured in all patients prior to DC therapy. The NLR was calculated and indicated that group A patients had a lower NLR than group B, C, and D patients (Figure 3A, 2.04 ± 0.48 versus 3.12 ± 1.34, 3.23 ± 1.04, and 3.43 ± 1.84, *p* = 0.032). Following ROC curve analysis, the area under the ROC for group A with objective response was 0.7908 (Figure 3B, 95% CI: 0.6768–0.9048, *p* = 0.003), and the best cut-off point prior to DC therapy was 2.835 (sensitivity: 1.000 and 1-specificity: 0.5667). When NLR was analyzed for all the patients with objective response and tumor regression, the area under the ROC curve was 0.6009 (Figure 3C, 95% CI: 0.4680–0.7338, *p* = 0.147). The best cut-off point was 2.935 (sensitivity: 0.6389 and 1-specificity: 0.5294).

### 2.5. T-Cell Frequency Alteration Following DC Immunotherapy

**Figure 4:**
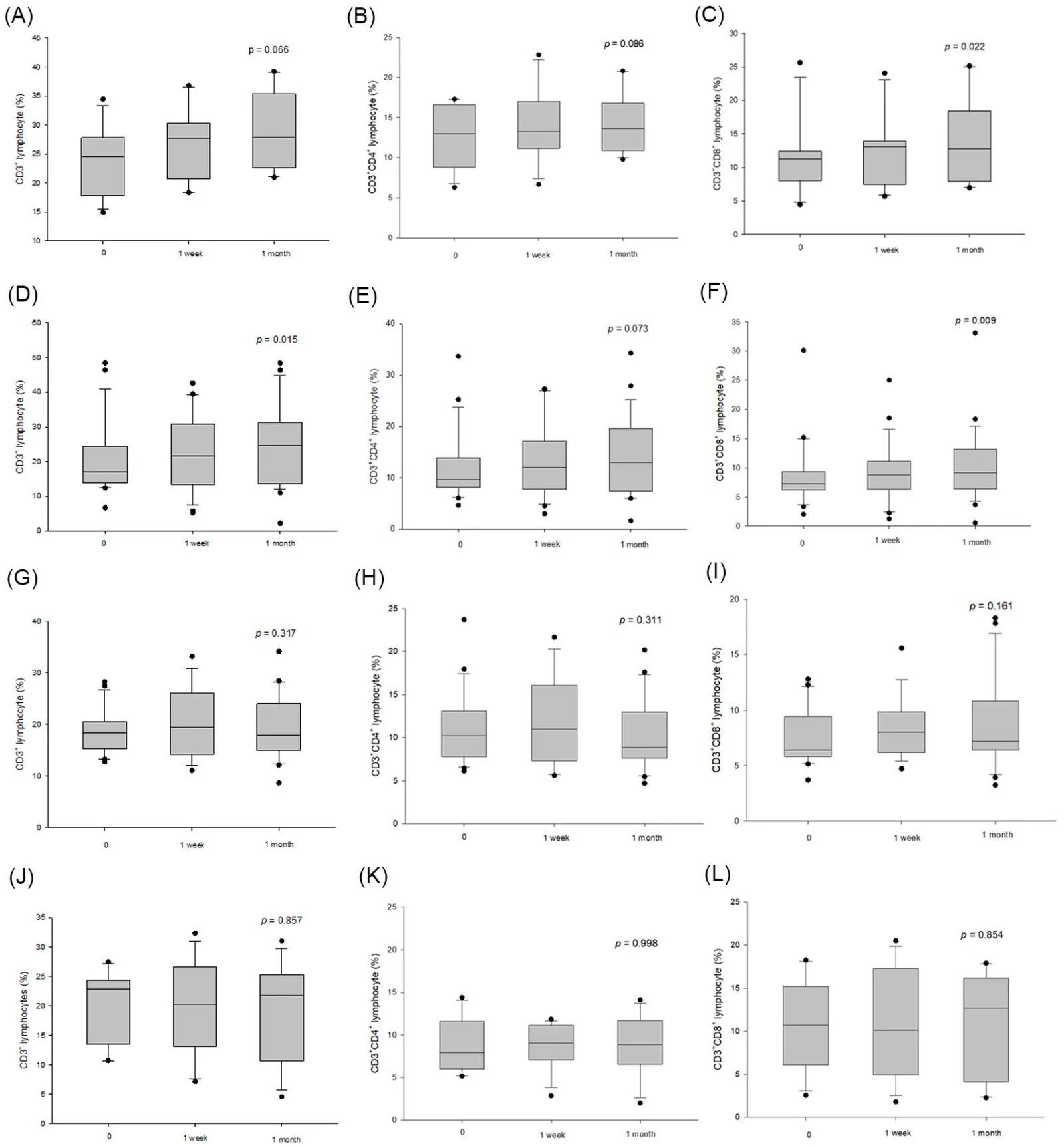
T-cell frequency alterations following DC immunotherapy. (**A**–**C**) For group A patients, the frequencies of CD3^+^ T-cells, CD4^+^ T-cells, and CD8^+^ T-cells showed a tendency to increase after DC immunotherapy. (**D**–**F**). For group B patients, the frequencies of CD3^+^ T-cells and CD8^+^ T-cells increased significantly, and the frequency of CD4^+^ T-cells also showed a tendency to increase after DC therapy. (**G**–**I**). For group C patients, the frequencies of total CD3^+^ T-cells, CD4^+^ T-cells, and CD8^+^ T-cells did not differ following DC immunotherapy. (**J**–**L**). For group D patients, the frequencies of total CD3^+^ T-cells, CD4^+^ T-cells, and CD8^+^ T-cells did not differ following DC immunotherapy.

T-cells in the peripheral blood were examined one week and one month after DC immunotherapy in the four groups of patients. For group A, the frequencies of CD3^+^ T-cells and CD4^+^ T-cells tended to increase after DC immunotherapy, and the median (interquartile) frequency of CD8^+^ T-cells increased significantly (from 11.3 (8.1–12.4)% to 13.1 (7.5–13.9)% at one week and 12.8 (7.9–18.4)% at one month, *p* = 0.022) (Figure 4A–C). For group B, the median (interquartile) frequencies of CD3^+^ T-cells increased significantly (from 17.0 (13.9–24.5)% to 21.8 (13.5–30.9)% at one week and 24.5 (13.6–31.3)% at one month, *p* = 0.015) and CD8^+^ T-cells (from 7.3 (6.2–9.3)% to 8.8 (6.3–11.2)% at one week and 9.2 (6.4–13.2)% at one month, *p* = 0.009), and the frequency of CD4^+^ T-cells also showed a tendency to increase after DC therapy (Figure 4D–F). For groups C and D, the frequencies of total CD3^+^ T-cells, CD4^+^ T-cells, and CD8^+^ T-cells before and after DC immunotherapy did not significantly differ (Figure 4G–L).

### 2.6. T-Cell Frequencies at One Month After DC Therapy

**Figure 5:**
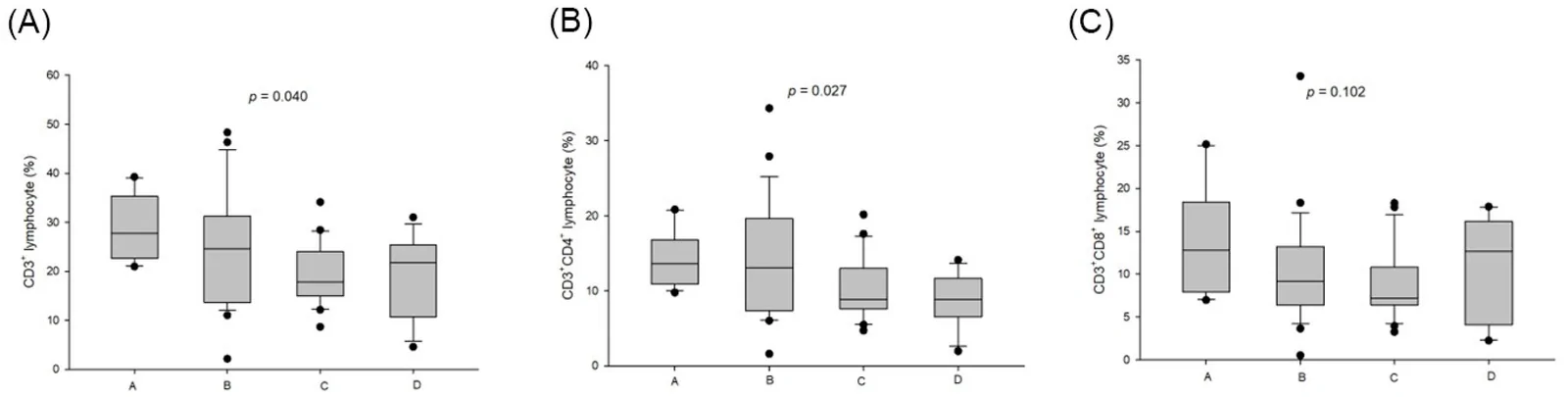
T-cell frequencies after DC therapy among the 4 groups (abscissae A to D). (**A**) After one month of DC therapy, the frequency of CD3^+^ T-cells was higher in group A than in the other groups (*p* = 0.040). (**B**) The frequency of CD4^+^ T-cells was higher in groups A and B than in groups C and D (*p* = 0.027). (**C**) The frequency of CD8^+^ T-cells did not differ among the 4 groups (*p* = 0.102).

After one month of DC therapy, the frequencies of T-cells were compared among the four groups. The median (interquartile) frequency of CD3^+^ T-cells was 27.8 (22.6–35.5)% for group A, 24.5 (13.6–31.3)% for group B, 17.9 (14.9–24.0)% for group C, and 21.7 (10.7–25.3)% for group D (*p* = 0.040). The median (interquartile) frequency of CD4^+^ T-cells was 13.7 (10.9–16.8)% for group A, 13.1 (7.4–19.6)% for group B, 8.9 (7.6–13.0)% for group C, and 8.9 (6.6–11.7)% for group D (*p* = 0.027). The median (interquartile) frequency of CD8^+^ T-cells was 12.8 (7.9–18.4)% for group A, 9.2 (6.4–13.2)% for group B, 7.2 (6.4–10.8)% for group C, and 12.7 (4.1–16.2)% for group D (*p* = 0.102) (Figure 5).

## 3. Discussion

Advanced-stage HCC is difficult to treat and is associated with poor prognosis. In recent years, the first-line treatment for advanced HCC has comprised a combination of ICI and TKI or dual ICI treatment [5,6,7]. The objective response rate is approximately 30%, and patients who achieve an objective response have significantly prolonged overall survival. Although these treatments show promising efficacy in advanced HCC, objective responses are achieved in only 30% of patients, leaving many patients with limited treatment options. Moreover, ICI-related adverse events may occur in up to 37% of patients [8], and life-threatening adverse effects may occur in 10% of patients. Taken together, these limitations have prompted the search for alternative or complementary treatment strategies for advanced HCC. DCs are the most potent antigen-presenting cells that activate antigen-specific T-cells. Autologous DC therapy has been used in the treatment of various cancers and has also been applied in patients with advanced HCC [16,17]. In this study, autologous DCs pulsed with tumor lysate were used as a supplemental therapy following ICI/TKI treatment for advanced HCC, resulting in an objective response rate of 15.7% and a minor regression rate of 37.1%. Patients who experience tumor regression, whether an objective response or minor regression, had better overall survival rates than those without tumor regression.

To our knowledge, immunity in early-stage cancers is not compromised as severely as that in late-stage cancers. Under DC immunotherapy following ICI/TKI, early-stage HCC may exhibit a better response than late-stage HCC. In this study, most of the patients were categorized as BCLC stage C, with only 10 patients categorized as late-stage B. Eight of the ten stage B patients had either an objective response or minor tumor regression, with five patients classified into group A and three into group B. From our findings, 80% of BCLC stage B patients responded well after DC immunotherapy. Liu et al. classified HCC patients into high- and low-immunity groups based on a single-sample gene set enrichment analysis score [18]. High-immunity patients had better overall survival than low-immunity patients, and the proportion of high-immunity patients was higher in the early stages than in the late stages. It was therefore not surprising that 8 of the 10 stage B patients exhibited tumor regression or minor responses after DC therapy in this study. These results also suggest that DC-based immunotherapy may be more effective at inducing tumor regression in earlier-stage disease than in advanced-stage cancer. For patients with advanced cancer, sufficient immunity preservation is crucial for effective anti-tumor immunity.

Lymphocytes mediate anti-cancer immunity, with T-cells playing an essential role in cancer cell eradication. T-cells are the downstream cells of DC therapy, and a high frequency of T-cells is crucial for DC-based immunotherapy. Therefore, we enrolled only patients with a lymphocyte frequency of ≥12%. Based on our findings, group A patients tended to have higher frequencies of total T-cells and CD8^+^ T-cells than patients in the other groups, although the difference in T-cell frequency did not reach statistical significance. After DC immunotherapy, patients with tumor responses in groups A and B had higher frequencies of total T-cells and CD4^+^ T-cells than those in groups C and D. In this study, tumor antigen delivery was achieved using a tumor lysate pulse during DC propagation. Antigens are expressed by DCs through an exogenous pathway. Notably, the frequency of CD4^+^ T-cells increased in patients in groups A and B. Although activated antigen-specific T-cells were not examined in this study, the increased frequencies of CD3^+^ T-cells and CD4^+^ T-cells may reflect increased anti-tumor immunity and were correlated with therapeutic efficacy.

The NLR is an effective indicator for predicting therapeutic responses to DC immunotherapy. As DC immunotherapy is a personalized and costly treatment, we sought to identify patients who would most benefit from DC-based immunotherapy. In this study, patients in group A exhibited a lower NLR than those in the other groups, and NLR ≤ 2.835 was identified as the best cut-off point to predict objective responses. The NLR has been recognized as a prognostic predictor in numerous cancers. In one study, patients with a low NLR had a better prognosis than those with a high NLR after hepatectomy for HCC [19]. In liver transplantation, patients with a low pre-transplant NLR had better outcomes [20]. Among patients with advanced HCC treated with a combination of atezolizumab and bevacizumab, those with disease control had a lower NLR than those with progressive disease, and an NLR ≤ 3.21 was identified as the cut-off value associated with better progression-free survival [21]. NLR reflects tumor-induced inflammation status and can be used as a reliable noninvasive marker to predict treatment efficacy. In this study, an NLR ≤ 2.835 was identified as a potential cut-off point for predicting an objective response to DC immunotherapy.

Surgical resection generally yields the most favorable results among all treatment modalities for HCC. In this study, all patients had advanced-stage disease; as such, surgery was not a viable option. After DC therapy, three patients in group A and one patient in group B experienced tumor regression and were converted to surgery, including liver resections and lung lobectomy. All four patients were tumor-free and survived well. The concept of conversion therapy from non-curative to curative treatment was recently highlighted by Dr. Kudo in the era of immunotherapy [22]. Patients in late-stage B or stage C cannot undergo curative surgery. In the era of immune checkpoint inhibitor therapy for HCC, tumor regression may occur in some patients, allowing their treatment to be converted to curative surgery. Supplemental DC therapy may induce tumor regression and, thus, the treatment can be converted to surgery in some patients after DC immunotherapy, as reported in this study. Locoregional therapy is another consideration after immunotherapy. Locoregional therapy for HCC may induce tumor cell necrosis and release tumor antigens to provoke anti-tumor immunity. Locoregional therapy may have a synergistic effect on immunotherapy or induce an abscopal effect.

The unique effects of DCs trigger antigen-specific immunity. Among patients with stable disease after DC therapy, the anti-tumor immune responses in those with minor regression and minor progression are likely to differ. Therefore, in this study, patients with stable disease were divided into two groups. T-cells were activated in group B but not in group C, and the overall survival rates differed substantially between the two groups. The patient survival rate in group B with minor regression approached that of group A, with 26.9% achieving 3-year survival, and was greater than that of the patients in group C with minor progression. The survival rate of the patients in group C was similar to that of group D. Therefore, provided anti-tumor immunity can be induced, prognosis may improve even when the tumor response is minimal.

For advanced HCC, the efficacy of single-modality treatment is limited. At present, the combination of an ICI and a TKI as well as dual ICIs are emerging therapies. However, the objective response rate remains approximately 30%, indicating that treatment efficacy could still be improved. Sequential or cocktail therapies may be suitable for such patients. In this study, DC immunotherapy was incorporated into the treatment strategy for advanced HCC, and 24 patients received sequential therapy with planned ICI followed by DC therapy. Eighteen (75%) patients experienced at least minor tumor regression and were stratified into groups A and B, with better outcomes than the other patients. Therefore, sequential therapy with scheduled ICI followed by DC therapy may be considered to improve therapeutic efficacy in patients with advanced HCC in the immunotherapy era. As the objective response of ICIs remains limited and DC therapy efficacy is impeded by immunosuppressor cells in the peripheral or tumor microenvironment, a combination of ICIs and DCs to deplete immunosuppressor cells and enhance anti-tumor immunity may represent another promising strategy for the treatment of advanced HCC. However, clinical trials are required to validate this concept.

DC therapy has been investigated as a treatment for advanced HCC for decades. However, clinical data remain limited because this treatment is personalized and requires specific facilities and technical expertise. This study was performed at a single center with selected patients. Regulatory T-cells, plasmacytoid DCs, and myeloid-derived immunosuppressor cells are well-established immunosuppressive cells associated with cancer prognosis. The phenotypes of these cells were examined using surface markers; however, their frequencies among the four groups did not differ. The changes in immune cells are dynamic, with various expressions. Immune cell examination at a fixed time during immunotherapy may not fully reflect true immune alterations. More detailed studies are required to differentiate patients’ immune status.

## 4. Materials and Methods

### 4.1. Patients

Seventy patients who received DC immunotherapy with immune cell analysis between 2020 and 2023 for BCLC (late stage B or stage C) were included in this study. We enrolled patients only if their lymphocyte frequency was ≥12% and excluded those patients with human immunodeficiency virus infection. The clinical profiles of the patients, previous treatments, and tumor responses after DC treatment were collected for biostatistical analysis. According to the tumor responses after DC therapy, the patients were divided into four groups: (A) patients with objective response (complete and partial response), (B) patients with stable disease and minor regression, (C) patients with stable disease and minor progression, and (D) patients with progressive disease. This DC-based treatment has been approved as a clinical treatment for advanced HCC by the Ministry of Health and Welfare, Taiwan, under special regulations. This study was designed to monitor immune alterations during DC-based immunotherapy, conformed to the ethical guidelines of the 2000 Declaration of Helsinki, and was approved by the Institutional Review Board of Chang-Gung Memorial Hospital, Linkou Main Branch (IRB No. 202000595B0). All patients provided written informed consent for DC-based immunotherapy and immune cell study.

### 4.2. Generation of DCs and DC Administration 

A sample of 50–60 mL of peripheral blood was obtained from each patient at each time point of DC propagation. Monocytes were isolated from the peripheral blood via Ficoll–Hypaque density centrifugation. The monocytes were then resuspended in serum-free AIM-V medium (Life Technologies, Carlsbad, CA, USA) and subsequently cultured for 9–10 days, supplemented with recombinant GM-CSF (1000 μ/mL; Schering-Plough, Kenilworth, NJ, USA) and IL-4 (1000 μ/mL; R&D System Inc., Minneapolis, MN, USA). Two days before DC harvesting, they were pulsed with autologous tumor lysates and matured with a cytokine cocktail, including IL-1b (10 ng/mL; R&D Systems Inc., Minneapolis, MN, USA), IL-6 (1000 μ/mL; 20 ng/mL; R&D Systems), TNF-a (20 ng/mL; R&D Systems), and PGE2 (1 mg/mL; Sigma, St. Louis, MO, USA) [23]. Before DC administration, the DCs must be negative for mycoplasma (performed via MycoTool Version 8mycoplasma real-time PCR), bacteria (performed via Gram stain, followed by analysis using a BD Bactec^TM^ Franklin Lakes, NJ, USA, FX blood culture system for 7 days), and endotoxin (performed using the Turbidimetric LAL method). The DCs were suspended in 5 mL normal saline and administered intravenously to the patients via a transfusion set over 5 min. The courses of DC therapy were administered at 2-week intervals.

### 4.3. Preparation of Tumor Lysates

Autologous tumor samples were obtained from the tumor biopsies or surgical specimens. Tumor cells were dispersed into a single-cell suspension (2 × 10^6^ cells/mL). Next, the cells were lysed by means of three cycles of snap freeze–thawing to obtain tumor lysates. Tumor lysis was monitored using light microscopy. Large particles were removed via centrifugation (5 min, 600 rpm). The supernatants were aliquoted and stored at −20 °C until use.

### 4.4. Analysis of Surface Molecular Expression on DCs

After directly staining the surface molecules with a panel of fluorescence-conjugated monoclonal antibodies, cell surface molecular expression on DCs was analyzed via cytofluorography using a Beckman Coulter NAVIOS flow cytometer (Beckman Coulter Co., Miami, FL, USA). The monoclonal antibodies used to assess the expression intensities of costimulatory molecules and MHC on DCs included phycoerythrin (PE)-conjugated mouse anti-CD40, CD54, CD80, CD83, CD86, and mouse anti-human anti-MHC class II (HLA-DR) (PharMingen, San Diego, CA, USA).

### 4.5. Analysis of Immune Cells

To assess changes in immune cells following DC immunotherapy, 1 mL of peripheral blood was obtained from each patient before DC therapy, 1 week after 3 courses of DC immunotherapy, and 1 month after DC therapy. Peripheral blood monocytes were stained with an antibody panel and analyzed via flow cytometry. Effector T-cells, regulatory T-cells, plasmacytoid DC, and myeloid-derived suppressor cells were examined. The antibody panel included CD3, CD4, CD8, CD11c, CD33, CD45, CD123, CCR7, Foxp3, and HLA-DR conjugated with FITC, PE, ECD, PC5.5, PC7, APC, AF647, AF700, APC-AF700, and APC-AF750 (DuraClone IM. antibody panel; Beckman Coulter, Brea, CA, USA).

### 4.6. Imaging Diagnosis of HCC

Diagnosis of HCC is based on multiphasic CT or MRI examination with contrast. The typical features of HCC are contrast uptake without a bright outer rim during the arterial phase and washout with a smooth and sharp rim enhancement during portal venous and delayed phases. In healthy livers, HCCs often present as large and dominant masses with well-defined margins. In cirrhotic livers, HCCs develop on a nodular and regenerating liver parenchymal background. Tumors tend to be smaller at detection, with poorly defined margins and heterogeneous enhancement. When the imaging findings demonstrate a typical pattern of HCC, liver biopsy is not required for diagnosis. Liver biopsy is reserved for liver tumors without a typical HCC pattern and when serum levels of AFP are not elevated.

### 4.7. Clinical Evaluation

Before DC immunotherapy, AFP levels, liver function tests for aspartate aminotransferase (AST) and alanine aminotransferase (ALT), hematological tests for hemoglobin, and differential white blood cell counts were performed. A computerized tomographic (CT) scan from the chest to the pelvis was performed to assess lung metastasis and peritoneal seeding and to measure tumor size, which was measured as the sum of the longest diameters of measurable tumors up to five tumors in total. Clinically complete response (CR) was defined as tumor disappearance. Partial response (PR) was defined as at least a 30% decrease in tumor size. Progressive disease (PD) was defined as >20% increase in tumor size or new tumor growth. Finally, stable disease (SD) was defined as disease between PR and PD lasting at least 6 weeks [24].

### 4.8. Biostatistics

All patients who received DC immunotherapy and had immune cell measurements were included in the analysis. Paired or non-paired Student’s t-tests were used to analyze continuous variables. Repeated measurements were analyzed via one-way repeated-measures ANOVA. Categorical variables were analyzed using either the chi-squared test or Fisher’s exact test. All pairwise multiple comparisons were performed using the Holm–Sidak method. A receiver operating characteristic (ROC) curve was generated to determine the best cut-off point. Survival was calculated from the dates of DC therapy. Survival rates were calculated using the Kaplan–Meier method and compared between the groups using the log-rank test. Statistical analyses were performed using SigmaPlot 16.0 software for Windows (Systat Software, Inc., San Jose, CA, USA). *p* < 0.05 was considered statistically significant.

## 5. Conclusions

DC immunotherapy can be applied as a supplemental treatment for advanced HCC in selected patients. Patients with T-cells provoked by DCs showed tumor regression and better outcomes. A low NLR prior to DC therapy can be used to predict tumor response after DC therapy.

## Figures and Tables

**Table 1:** Characteristics of 70 patients receiving DC immunotherapy for advanced HCC.

	Group A (n = 11)	Group B (n = 26)	Group C (n = 21)	Group D (n = 12)	*p*
Age (years) Median (interquartile)	59 (46–67)	59 (55–64)	55 (49–70)	54 (45–64)	0.340
Sex (male/female)	9/2	19/7	17/4	9/3	0.900
BCLC stage (B/C)	5/6	3/23	1/20	1/11	0.013
TNM stage (2/3/4)	3/4/4	2/11/13	1/5/15	1/2/9	0.190
Child–Pugh classification (A/B)	11/0	25/1	21/0	11/1	0.507
Viral hepatitis (non/B/C/B+C)	0/9/2/0	0/22/3/1	3/15/2/1	3/8/0/1	0.263
TKI (+/−) prior to DCa	7/4	22/4	18/3	7/5	0.228
Sorafenib	3	8	8	3	
Sorafenib + Regorafenib	1	2	4	2	
Lenvatinib	3	12	6	2	
ICI (+/−) prior to DCs	7/4	16/10	14/7	7/5	0.643
Nivolumab	2	3	4	2	
Pembrolizumab	5	11	8	2	
Durvalumab	0	1	2	0	
Atezolizumab + bevacizumab	0	1	0	3	
Planned ICI before DCs (+/−)	6/1	12/4	3/11	3/4	0.007
* Number of DCs administered Median (interquartile) (×10^6^)	16.72 (12.13–28.30)	16.33 (10.42–28.58)	15.73 (11.94–25.13)	17.54 (15.31–21.86)	0.962

DC, dendritic cell; HCC, hepatocellular carcinoma; BCLC, Barcelona Clinic Liver Cancer classification; TKI, tyrosine kinase inhibitor; ICI, immune checkpoint inhibitor; TNM, tumor–lymph node–metastasis classification. * The total number of DCs in three applications.

**Table 2:** Immune cell counts in peripheral blood prior to DC immunotherapy according to tumor responses.

Cell Type	Group A (n = 11)	Group B (n = 26)	Group C (n = 21)	Group D (n = 12)	*p*
CD3^+^ (%)	24.5 (17.8–27.8)	17.0 (13.9–24.5)	18.30 (15.3–20.6)	22.9 (13.6–24.4)	0.086
CD3^+^CD4^+^ (%)	13.0 (8.8–16.6)	9.6 (8.2–13.9)	10.2(7.8–13.1)	7.9 (6.0–11.2)	0.131
CD3^+^CD8^+^ (%)	11.3 (8.1–12.4)	7.3 (6.2–9.3)	6.4 (5.8–9.4)	10.7 (6.1–15.2)	0.064
CD4^+^ Foxp3^+^ (%)	8.76 (5.59–11.98)	6.27 (4.83–10.27)	5.84 (4.06–8.70)	5.55 (4.73–8.49)	0.375
HLA DR^+^ (%)	7.0 (6.1–11.1)	8.7 (2.7–10.7)	7.3 (5.4–9.8)	10.8 (5.4–13.0)	0.600
CD11c^−^CD123^+^ in HLA DR+ (%)	18.2 (2.9–33.8)	11.3 (5.1–18.3)	4.3 (2.3–11.8)	15.5 (3.7–20.0)	0.148
CD11c^+^CD123^−^ in HLA DR^+^ (%)	23.1 (11.5–62.7)	23.4 (2.1–34.3)	20.6 (4.2–37.9)	17.4 (5.9–25.0)	0.623
HLA DR^−^CD33^+^ (%)	1.61 (0.24–2.29)	1.57 (1.04–2.17)	1.97 (1.14–3.20)	1.65 (0.5–3.18)	0.691

Data are presented as median (interquartile).

## Data Availability

The original contributions presented in this study are included in the article. Further inquiries can be directed to the corresponding author.

## Abbreviations

DC	dendritic cell
HCC	hepatocellular carcinoma
ICI	immune checkpoint inhibitor
irAE	immune-related adverse effect
NLR	neutrophil-to-lymphocyte ratio
PBMC	peripheral blood monocyte
TKI	tyrosine kinase inhibitor
